# Identification of SERPINE1, PLAU and ACTA1 as biomarkers of head and neck squamous cell carcinoma based on integrated bioinformatics analysis

**DOI:** 10.1007/s10147-019-01435-9

**Published:** 2019-04-01

**Authors:** Ke Yang, Shizhou Zhang, Dongsheng Zhang, Qian Tao, Tianqi Zhang, Guijun Liu, Xingguang Liu, Tengda Zhao

**Affiliations:** 1 Department of Oral and Maxillofacial Surgery, Provincial Hospital Affiliated to Shandong University, Jinan, 250021 Shandong China; 2 Department of Medical Center, Provincial Hospital Affiliated to Shandong University, Jinan, Shandong China; 3 Department of Oral and Maxillofacial Surgery, Guanghua School of Stomatology, Hospital of Stomatology, Sun Yat-Sen University, Guangdong Provincial Key Laboratory of Stomatology, Guangzhou, Guangdong China; 4 Shangdong Provincial Key Laboratory of Oral Tissue Regeneration, Stomatology Hospital of Shandong University, Jinan, Shandong China

**Keywords:** HNSCC, Biomarkers, Differentially expressed genes, Integrated bioinformatics analysis

## Abstract

**Background:**

Head and neck squamous cell carcinoma (HNSCC) is the six leading cancer by incidence worldwide. The 5-year survival rate of HNSCC patients remains less than 65% due to lack of symptoms in the early stage. Hence, biomarkers which can improve detection of HNSCC should improve clinical outcome.

**Methods:**

Gene expression profiles (GSE6631, GSE58911) and the Cancer Genome Atlas (TCGA) HNSCC data were used for integrated bioinformatics analysis; the differentially expressed genes (DEGs) were then subjected to functional and pathway enrichment analysis, protein–protein interaction (PPI) network construction. Subsequently, module analysis of the PPI network was performed and overall survival (OS) analysis of hub genes in subnetwork was studied. Finally, immunohistochemistry was used to verify the selected markers.

**Results:**

A total of 52 up-regulated and 80 down-regulated DEGs were identified, which were mainly associated with ECM–receptor interaction and focal adhesion signaling pathways. Importantly, a set of prognostic signatures including SERPINE1, PLAU and ACTA1 were screened from DEGs, which could predict OS in HNSCC patients from TCGA cohort. Experiment of clinical samples further successfully validated that these three signature genes were aberrantly expressed in the oral epithelial dysplasia and HNSCC, and correlated with aggressiveness of HNSCC patients.

**Conclusions:**

SERPINE1, PLAU and ACTA1 played important roles in regulating the initiation and progression of HNSCC, and could be identified as key biomarkers for precise diagnosis and prognosis of HNSCC, which will provide potential targets for clinical therapies.

**Electronic supplementary material:**

The online version of this article (10.1007/s10147-019-01435-9) contains supplementary material, which is available to authorized users.

## Introduction

Head and neck squamous cell carcinoma (HNSCC), which arises from the oral cavity, larynx and pharynx, ranks as the sixth most common malignancy with an estimated 835,000 new cases and 43,000 associated deaths worldwide in 2018 [1, 2]. Unfortunately, diagnosis of HNSCC is usually made at advanced stages due to lack of symptoms in the early stage of head and neck tumorigenesis, and the 5-year survival rate is still less than 65% now [3]. It is widely believed that accumulation of numerous genetic alterations in epithelial cells is the essential process driven by the initiation and progression of HNSCC [4]. Therefore, investigation of the potential key biomarkers may help to further uncover the biological basis of HNSCC and improve clinical therapy.

Recently, microarrays based on high-throughput platforms for analysis of gene expression are increasingly valued as a promising and efficient tool to screen significant genetic alternations in carcinogenesis and identify biomarkers for diagnosis and prognosis of cancer [5]. A number of gene expression profiling microarrays have been conducted to find various differentially expressed genes (DEGs) in HNSCC [6]; however, considerable efforts in the identification of biomarker have met with limited success, primarily because of independent numbers of gene profiling. Now, by the means of integrated bioinformatics analysis for available microarray data, it is possible to make more reliable and precise screening results via overlapping relevant datasets.

In the current study, microarray data of gene expression profiles (GSE6631 [7], GSE58911 [8]) and the Cancer Genome Atlas (TCGA) HNSCC data [9] were integrated and analyzed by a series of biological informatics approaches, aberrantly DEGs and pathways were identified in HNSCC, protein–protein interaction (PPI) network was also constructed and hub genes were revealed. Subsequently, we investigated the relationship between the hub genes of subnetwork and overall survival (OS) in TCGA database, and tested the expression status of these hub genes in clinical samples at different stages of tumorigenesis. By this mean, we may bring to light the underlying mechanisms and identify the potential candidate biomarkers for diagnosis and prognosis of HNSCC.

## Materials and methods

### Microarray data

In the present study, the gene expression profiles (GSE6631, GSE58911) were obtained from Gene Expression Omnibus (GEO, https://www.ncbi.nlm.nih.gov/geo/). Totally 22 paired samples of HNSCC and normal tissues were consisted in GSE6631, which based on GPL8300 platform (Affymetrix Human Genome U95 chips). GSE58911 dataset was already deposited in GPL6244 (Affymetrix Human Gene 1.0 ST Array), including 15 paired normal and HNSCC samples. Moreover, the TCGA HNSCC data (https://cancergenome.nih.gov/) were also downloaded, including 44 normal and 502 HNSCC tissues. We chose these 3 datasets for integrated analysis to identify commonly DEGs.

### Data processing and identification of DEGs

The original raw array data were subjected to background correction, quartile data normalization, and converted into gene expression values. Data were normalized using the Bioconductor R package (https://cran.r-project.org/mirrors.html). Then, the DEGs between HNSCC samples and normal controls were identified using the empirical Bayes approach in linear models for the microarray data (limma) package. |logFC| > 1 and *p* < 0.05 were selected as the cutoff criterion.

### Functional and pathway enrichment analysis of DEGs

To analyze the identified DEGs at the functional level, the significant gene ontology (GO) biological process terms [10] and Kyoto Encyclopedia of Genes and Genomes (KEGG) pathway enrichment analysis [11] were performed using the Database for Annotation, Visualization and Integrated Discovery (DAVID, https://david.ncifcrf.gov/) with the thresholds of *p* < 0.05 and false discovery rate (FDR) < 0.01 [12].

### Modules from the PPI network

To evaluate the interactive relationships among DEGs, we mapped the DEGs to the Search Tool for the Retrieval of Interacting Genes (STRING) database (https://string-db.org) [13]. Then, the interactive DEGs were selected to construct the PPI network (combined score ≥ 0.4) and visualized using Cytoscape [14]. The Molecular Complex Detection (MCODE) plugin in Cytoscape was used to screen the modules of PPI network with MCODE score > 3 and number of nodes > 4.

### Survival analysis of the hub gene in TCGA database

The association between the corresponding genes in the top modules and patient OS for 5 years was analyzed using HNSCC samples from the TCGA data. All HNSCC patients were classified into high or low expression based on whether *Z*-score expression was > median (high) or < median (low), log-rank analysis and Kaplan–Meier plots were produced using Bioconductor R package.

### Clinical samples and clinical staging system

A total of 52 paraffin-embedded HNSCC (39) and oral epithelial dysplasia (OED, 13) samples were obtained from the archives of the Department of Pathology of Shandong Provincial Hospital, Jinan, China. Of the HNSCC samples, there were 23 (59%) well, 10 (26%) moderately and 6 (15%) poorly differentiated HNSCC tissues; 10 matched adjacent non-cancerous oral mucosa (NOM) tissues were selected from the above-mentioned patients and detailed sample information is presented in Supplementary Table 1. For the use of these clinical materials for research purposes, prior patient consent and approval from the Institute Research Ethics Committee were obtained. The approval number was no. 2018-037. The stages of all HNSCC patients were classified according to the Union for International Cancer Control (UICC 2017).

### Immunohistochemical analysis

Protein expression was evaluated on paraffin-embedded sections using microwave antigen retrieval with 0.01 M citrate buffer (pH 6.0). Rabbit polyclonal anti-SERPINE1 (1:200, sc-5297, Santa Cruz), rabbit polyclonal anti-PLAU (1:150, ab133563, Abcam) and rabbit polyclonal anti-ACTA1 (1:100, sc-5867, Santa Cruz) antibodies were utilized. The immunohistochemical procedure was as previously described [15, 16]. Interpretation of immunohistochemical staining was made independently by two specialists and the mean protein staining intensity (SI; 0, no staining; 1, weak; 2, moderate; 3, strong), labeling indices (LIs, defined as the percentage of positive cells in total cells), and mean labeling scores (LS, defined as LI × SI) in HNSCC, OED and NOM samples were calculated and compared among groups.

### Statistical analysis

Comparison of two Kaplan–Meier curves was performed using the log-rank test of the R-package survival. The mean LS for HNSCC, OED and NOM samples was compared among the three groups by analysis of variance (ANOVA) using the SPSS 10.0 software package. *p* < 0.05 was considered statistically significant.

## Results

### Identification of aberrantly DEGs in HNSCC

**Fig. 1:**
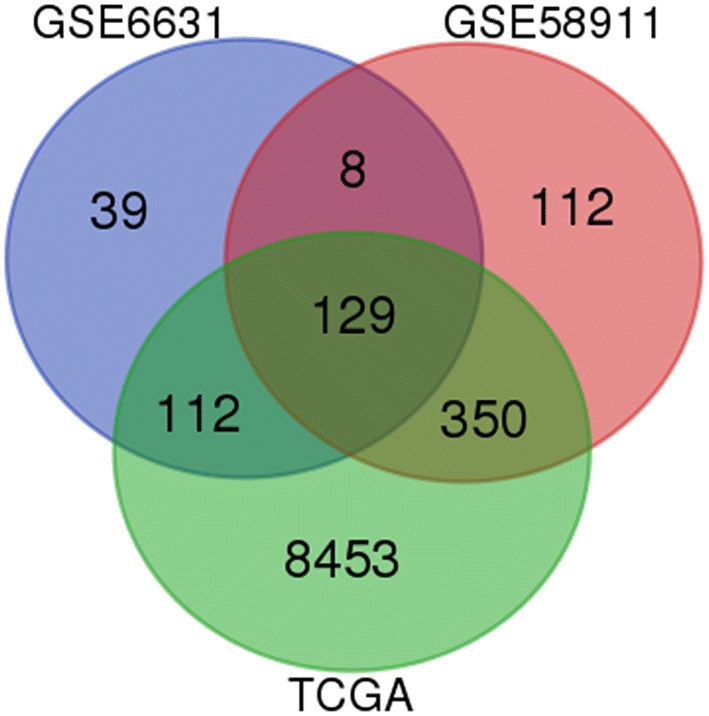
Identification of 132 common DEGs from the three cohort profile datasets (GSE6631, GSE58911 and TCGA) using Morpheus Website (https://software.broadinstitute.org). Different color areas represented different datasets. The cross areas meant the commonly changed DEGs. DEGs were identified with classical t test; statistically significant DEGs were defined with *p* < 0.05 and |logFC| > 1 as the cutoff criterion

Data from each microarray were separately analyzed to screen DEGs. As represented in Fig. 1, our integrated bioinformatics analysis indicated that a total of 132 genes were consistently and significantly deregulated in the same direction in these datasets, including 52 overlapping up-regulated (163 in GSE6631, 172 in GSE58911 and 5679 in TCGA) and 80 down-regulated genes (128 in GSE6631, 427 in GSE58911 and 3436 in TCGA) in HNSCC tissues, as compared to normal epithelial tissues (Table 1).

**Table 1.** 132 DEGs were identified from three profile datasets, including 52 up-regulated genes and 80 down-regulated genes in the HNSCC tissues, compared to normal tissue

DEGs	Gene names
Up-regulated	PXDN, KIF14, CDH11, NELL2, MMP1, FCGR2A, MMP3, FAT1, NREP, LAPTM4B, MMP13, PTHLH, MYO10, DFNA5, COL3A1, BST2, COL6A3, SPARC, PRAME, RBP1, IGF2BP3, LTBP1, FN1, LAMB3, COL5A2, SPP1, ITGA6, PLOD2, MMP12, MMP11, COL4A2, LUM, POSTN, MFAP2, COL4A1, LOX, LAMC2, MMP10, SLC16A1, PLAU, CXCL8, PFN2, TNC, FAP, LAMB1, LOXL2, DLX5, SERPINE1, COL10A1, TGFBI, SEMA3C, MMP9
Down-regulated	CEACAM6, ENDOU, TF, MYH2, FHL1, CSRP3, SORBS2, SPINK5, CSTA, PPL, PITX1, CLDN10, MALL, GPD1L, PDK4, BLNK, ALOX12, SASH1, KRT4, AQP3, FUT3, APOD, DIO2, IL1RN, SLURP1, ACTA1, CEACAM1, CKMT2, LPIN1, PSCA, SULT2B1, KAT2B, ABLIM1, HOPX, PTN, AADAC, KRT13, CRYAB, MAL, ECM1, ANXA9, EMP1, ATP2A1, ABCA8, SERPINB2, ADH7, FCER1A, TGM3, MYL1, ACPP, HPGD, SCEL, MYLPF, CRISP3, NEB, TGM1, CD24, NUCB2, KLK13, MB, EXPH5, SERPINB1, ALDH3A1, CXCL12, MYH7, FMO2, ZNF185, TTN, FLG, ADH1B, COX6A2, PDLIM3, PPP1R3C, RRAGD, LCN2, CASQ2, KLK11, TTC9, PGD, ATP10B

### DEGs functional and pathway enrichment analysis

The top 5 significant terms of GO analysis in DAVID were illustrated in Table 2. In the biological process (BP) group, GO analysis results showed that up-regulated DEGs were significantly enriched in extracellular matrix organization, collagen catabolic process, extracellular matrix disassembly, cell adhesion and collagen fibril organization; the down-regulated DEGs were mainly enriched in muscle contraction and muscle filament sliding. For molecular function (MF), the enrichment of up-regulated DEGs was in metalloendopeptidase activity, extracellular matrix structural constituent, serine-type endopeptidase activity, collagen binding and endopeptidase activity, and down-regulated genes were enriched in structural constituent of muscle. Besides, GO cell component (CC) analysis indicated that the enrichment of up-regulated DEGs was predominantly in extracellular matrix, proteinaceous extracellular matrix, extracellular region, extracellular space and basement membrane, and down-regulated DEGs were enriched in extracellular exosome, muscle myosin complex and Z disc.

**Table 2:** Go analysis of DEGs associated with HNSCC

Category	Term	Count	%	*p* value	FDR
Up-regulated DEGs					
GOTERM_BP	GO:0030198 ~ extracellular matrix organization	21	40.38	4.57E−26	6.38E−23
GOTERM_BP	GO:0030574 ~ collagen catabolic process	13	25	4.42E−19	6.17E−16
GOTERM_BP	GO:0022617 ~ extracellular matrix disassembly	11	21.15	2.15E−14	3.01E−11
GOTERM_BP	GO:0007155 ~ cell adhesion	14	26.92	7.43E−10	1.04E−06
GOTERM_BP	GO:0030199 ~ collagen fibril organization	6	11.54	1.13E−07	1.57E−04
GOTERM_CC	GO:0031012 ~ extracellular matrix	19	36.54	6.18E−20	6.78E−17
GOTERM_CC	GO:0005578 ~ proteinaceous extracellular matrix	18	34.62	3.89E−19	4.27E−16
GOTERM_CC	GO:0005576 ~ extracellular region	30	57.69	4.60E−18	5.05E−15
GOTERM_CC	GO:0005615 ~ extracellular space	24	46.15	2.09E−13	2.30E−10
GOTERM_CC	GO:0005604 ~ basement membrane	7	13.46	8.44E−08	9.25E−05
GOTERM_MF	GO:0004222 ~ metalloendopeptidase activity	8	15.38	3.40E−08	3.80E−05
GOTERM_MF	GO:0005201 ~ extracellular matrix structural constituent	7	13.46	8.80E−08	4.25E−05
GOTERM_MF	GO:0004252 ~ serine-type endopeptidase activity	9	17.31	6.41E−07	7.17E−04
GOTERM_MF	GO:0005518 ~ collagen binding	6	11.54	8.09E−07	9.05E−04
GOTERM_MF	GO:0004175 ~ endopeptidase activity	5	9.62	1.78E−05	1.99E−03
Down-regulated DEGs					
GOTERM_BP	GO:0006936 ~ muscle contraction	8	10	5.33E−07	7.80E−04
GOTERM_BP	GO:0030049 ~ muscle filament sliding	6	7.5	7.42E−07	1.09E−03
GOTERM_CC	GO:0070062 ~ extracellular exosome	34	42.5	1.66E−08	1.95E−05
GOTERM_CC	GO:0005859 ~ muscle myosin complex	5	6.25	5.72E−07	6.73E−04
GOTERM_CC	GO:0030018 ~ Z disk	8	10	7.87E−07	9.26E−04
GOTERM_CC	GO:0005615 ~ extracellular space	20	25	3.46E−06	4.07E−03
GOTERM_MF	GO:0008307 ~ structural constituent of muscle	7	8.75	3.40E−08	4.25E−05

We also determined the canonical signaling pathways associated with the commonly DEGs in the carcinogenesis of HNSCC by performing KEGG analysis. The activated pathways were enriched in ECM–receptor interaction, focal adhesion, PI3K–Akt signaling pathway, while the suppressed pathways were mainly involved in drug metabolism–cytochrome P450, tyrosine metabolism and tight junction (Table 3).

**Table 3:** KEGG pathway enrichment analysis of up-regulated and down-regulated DEGs

Pathways	Name	Count	%	*p* value	Genes
Up-regulated DEGs					
hsa04512	ECM–receptor interaction	12	23.08	7.28E−15	COL4A2, LAMB3, COL4A1, ITGA6, TNC, COL3A1, COL6A3, LAMC2, LAMB1, COL5A2, SPP1, FN1
hsa04510	Focal adhesion	12	23.08	1.09E−10	COL4A2, LAMB3, COL4A1, ITGA6, TNC, COL3A1, COL6A3, LAMC2, LAMB1, COL5A2, SPP1, FN1
hsa05146	Amoebiasis	9	17.31	4.10E−09	COL4A2, LAMB3, COL4A1, COL3A1, CXCL8, LAMC2, LAMB1, COL5A2, FN1
hsa04151	PI3K–Akt signaling pathway	12	23.08	2.60E−08	COL4A2, LAMB3, COL4A1, ITGA6, TNC, COL3A1, COL6A3, LAMC2, LAMB1, COL5A2, SPP1, FN1
hsa05222	Small cell lung cancer	7	13.46	6.98E−07	COL4A2, LAMB3, COL4A1, ITGA6, LAMC2, LAMB1, FN1
hsa05200	Pathways in cancer	10	19.23	1.06E−05	COL4A2, LAMB3, COL4A1, ITGA6, MMP9, CXCL8, LAMC2, LAMB1, MMP1, FN1
hsa04974	Protein digestion and absorption	6	11.54	1.93E−05	COL4A2, COL4A1, COL3A1, COL6A3, COL5A2, COL10A1
hsa05145	Toxoplasmosis	4	7.69	0.0105	LAMB3, ITGA6, LAMC2, LAMB1
hsa05219	Bladder cancer	3	5.77	0.0110	MMP9, CXCL8, MMP1
hsa05202	Transcriptional misregulation in cancer	4	7.69	0.0269	MMP9, CXCL8, MMP3, PLAU
hsa05205	Proteoglycans in cancer	4	7.69	0.0419	LUM, MMP9, PLAU, FN1
hsa05323	Rheumatoid arthritis	3	5.77	0.0458	CXCL8, MMP3, MMP1
Down-regulated DEGs					
hsa00982	Drug metabolism-cytochrome P450	4	5	0.0040	FMO2, ADH1B, ADH7, ALDH3A1
hsa00350	Tyrosine metabolism	3	3.75	0.0119	ADH1B, ADH7, ALDH3A1
hsa04530	Tight junction	4	5	0.0270	MYH2, MYLPF, CLDN10, MYH7
hsa00010	Glycolysis/gluconeogenesis	3	3.75	0.0403	ADH1B, ADH7, ALDH3A1
hsa00980	Metabolism of xenobiotics by cytochrome P450	3	3.75	0.0483	ADH1B, ADH7, ALDH3A1

### PPI network construction and module analysis

**Fig. 2:**
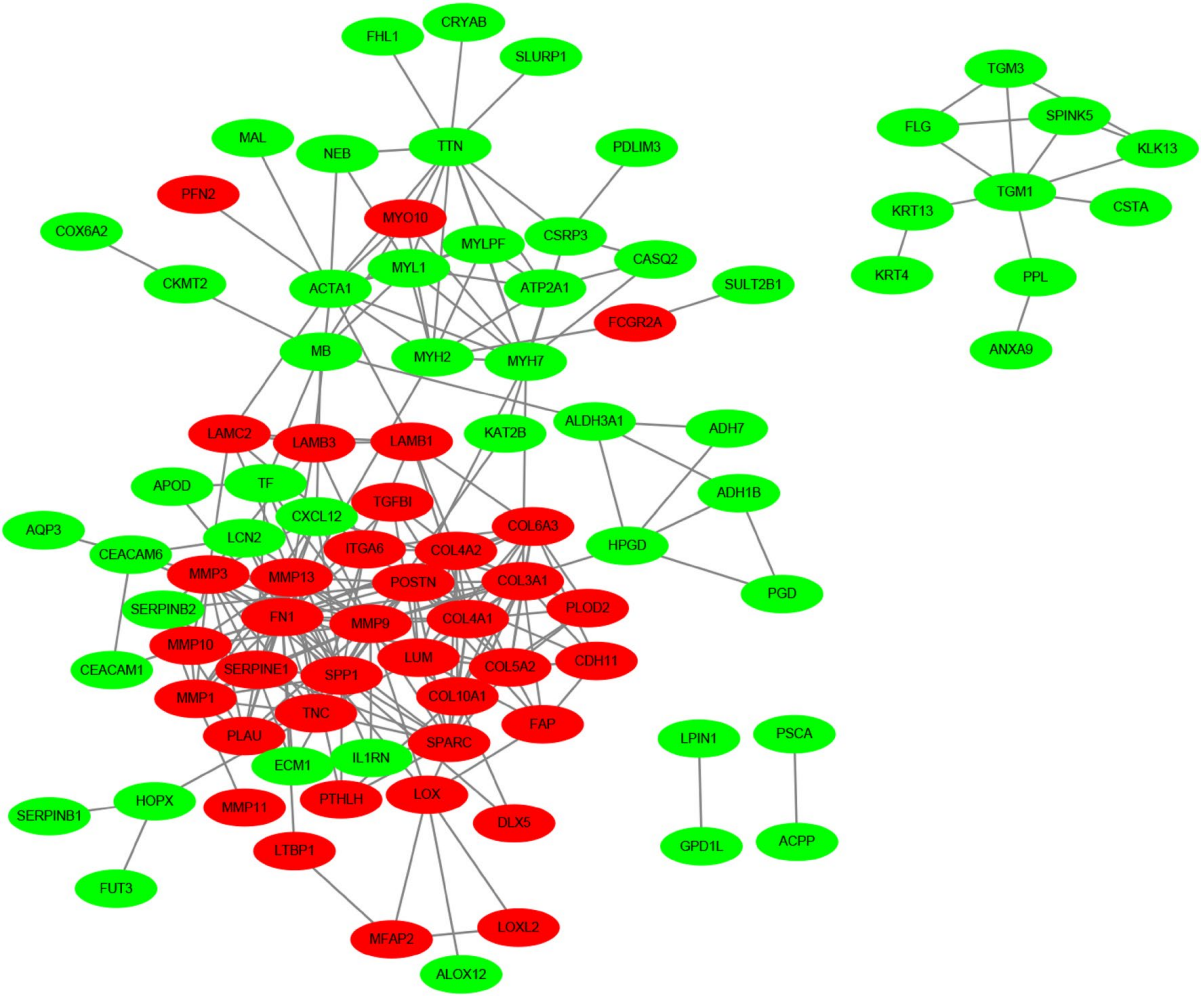
DEGs PPI network complex and module analysis. Using the STRING online database, a total of 90 DEGs (37 up-regulated in red standing and 53 down-regulated genes in green standing) were filtered into the DEGs PPI network complex

**Fig. 3:**
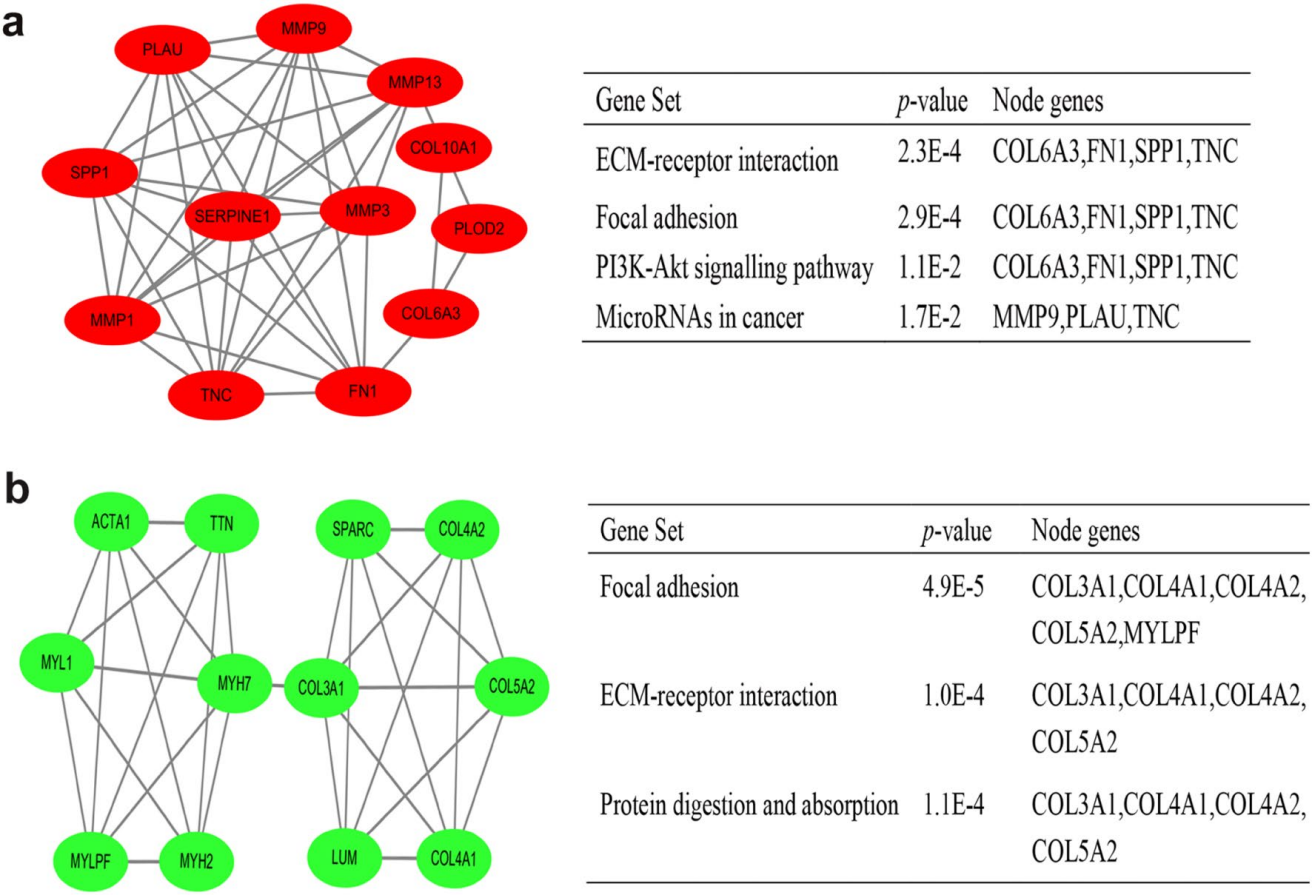
Top two modules from PPI network. **a** Module 1 and its enriched pathways (upper); **b** module 2 and its enriched pathways (bottom)

Using the STRING online database and Cytoscape software, 90 DEGs (37 up-regulated and 53 down-regulated genes) of the 132 commonly DEGs were filtered into the PPI network complex, containing 131 nodes and 289 edges (Fig. 2), and the top 10 node degree genes were FN1, MMP9, SPP1, COL3A1, MMP13, POSTN, SPARC, COL4A1, ACTA1 and SERPINE1. According to the importance of degree, we chose two most significant modules from the PPI network complex for further analysis using Cytoscape MCODE. Pathway enrichment analysis showed that the module 1 consisted of 12 nodes and 40 edges, which were mainly associated with ECM–receptor interaction, focal adhesion and PI3K–Akt signaling pathway, while the module 2 included 12 nodes and 31 edges, which were also enriched in focal adhesion and ECM–receptor interaction (Fig. 3), suggesting that ECM–receptor interaction and focal adhesion signaling pathways were essential in the carcinogenesis of HNSCC.

### The validation of hub genes as independent predictors for OS in the TCGA database

**Fig. 4:**
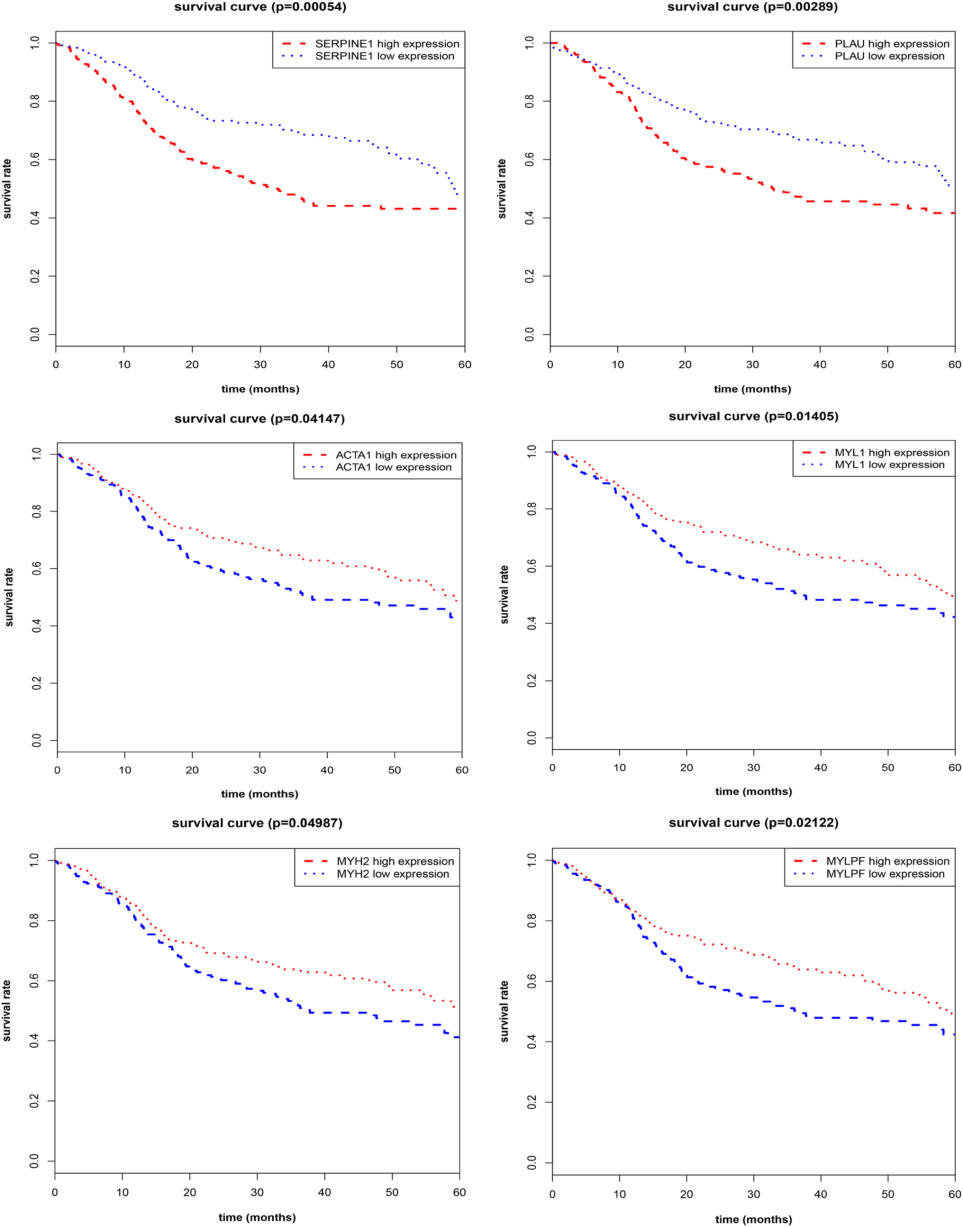
Kaplan–Meier curves exhibit the OS in the TCGA HNSCC cohort with high and low expression of SERPINE1, PLAU, ACTA1, MYL1, MYH2 and MYLPF

We subsequently sought to assess the significance of hub genes for HNSCC; the relationships between expression of hub genes and OS were verified in the TCGA HNSCC cohort. For most of the hub genes, our results showed that poor OS was associated only in those patients with high expression of SERPINE1 (*p* = 0.00054) or PLAU (*p* = 0.00289), as well as the low expression of ACTA1 (*p* = 0.04147), MYL1 (*p* = 0.01405), MYH2 (*p* = 0.04987) or MYLPF (*p* = 0.02122) (Fig. 4), suggesting that these candidate genes are associated with clinical outcome of HNSCC patients.

### SERPINE1, PLAU and ACTA1 are aberrantly expressed in the carcinogenesis of HNSCC.

**Fig. 5:**
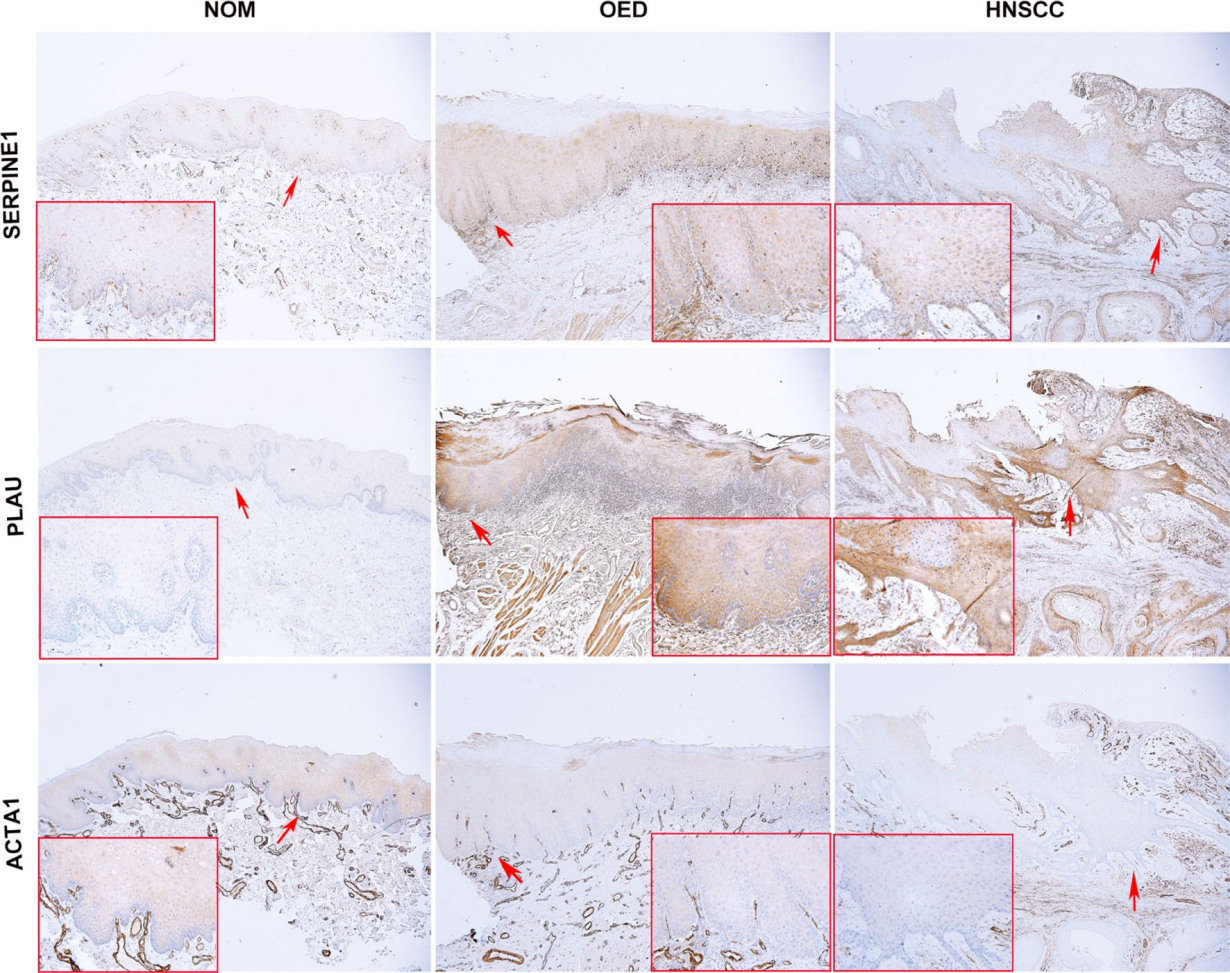
Immunohistochemical staining of SERPINE1, PLAU and ACTA1 in oral epithelium at different stages of head and neck carcinogenesis (× 40). The SERPINE1 was expressed in nucleus and cytoplasm, while PLAU was mainly expressed in cytoplasm; the cytoplasmic and/or nuclear staining intensity of basal layers were much denser than that of upper epithelial cells for SERPINE1 and PLAU. The expression of ACTA1 was mainly distributed at the cytoplasm of epithelial cells in prickle layer (red arrow, × 200). NOM, non-cancerous oral mucosa; OED, oral epithelial dysplasia

To further clarify the potential biological role of these prognosis-associated genes in HNSCC transformation, we next characterized the expression changes of signature genes by microarray analysis (Supplementary Fig. 1). Among these hub genes that had large change levels in HNSCC samples, we compared their degrees in the highest ranked modules, two up-(SERPINE1, PLAU) and one down-regulated (ACTA1) genes were particularly selected to further test the protein expression in NOM, OED and HNSCC tissues. As expected, we found that the expression of SERPINE1 and PLAU increased from NOM to OED and HNSCC. The mean LS of SERPINE1 increased in OED (230.38 ± 53.046%) and HNSCC samples (205.85 ± 51.427%), compared with that of NOM (58.50 ± 15.072%). Likewise, the level of PLAU also increased significantly from NOM (51.80 ± 10.727%) through OED (194.54 ± 46.321%) to HNSCC samples (199.61 ± 47.895%), there was a significant difference in the mean LS of SERPINE1 or PLAU between NOM and OED (*p* = 0.000) or HNSCC samples (*p* = 0.000), respectively; however, the LS of SERPINE1 or PLAU between OED and HNSCC had no significant difference. Representative microphotographs of SERPINE1 and PLAU staining for NOM, OED and HNSCC are shown in Fig. 5. Instead, the level of ACTA1 showed an opposite trend; the LS was reduced from NOM (136.10 ± 49.249%) through OED (81.77 ± 5.403%) to HNSCC samples (60.66 ± 9.089%). There was a significant difference in the expression of ACTA1 among the 3 groups (Table 4). Thus, combined with the TCGA data analysis, these results suggested that SERPINE1, PLAU and ACTA1 are required for the initiation of head and neck tumorigenesis.

**Table 4:** The mean LS of SERPINE1, PLAU and ACTA1 in NOM, OED and HNSCC tissues

Group	Mean LS ± SD (100%)		
	SERPINE1	PLAU	ACTA1
NOM	58.50 ± 15.072	51.80 ± 10.727	136.10 ± 49.249
OED	230.38 ± 53.046*	194.54 ± 46.321*	81.77 ± 5.403*
HNSCC	205.85 ± 51.427*	199.61 ± 47.895*	60.66 ± 9.089*^,^^#^

*LS* labeling scores, *NOM* non-cancerous oral mucosa, *OED* oral epithelial dysplasia

*p < 0.05, compared to NOM group; ^#^*p *< 0.05, compared to OED group

### SERPINE1, PLAU and ACTA1 are correlated with clinical aggressiveness of HNSCC patients

**Fig. 6:**
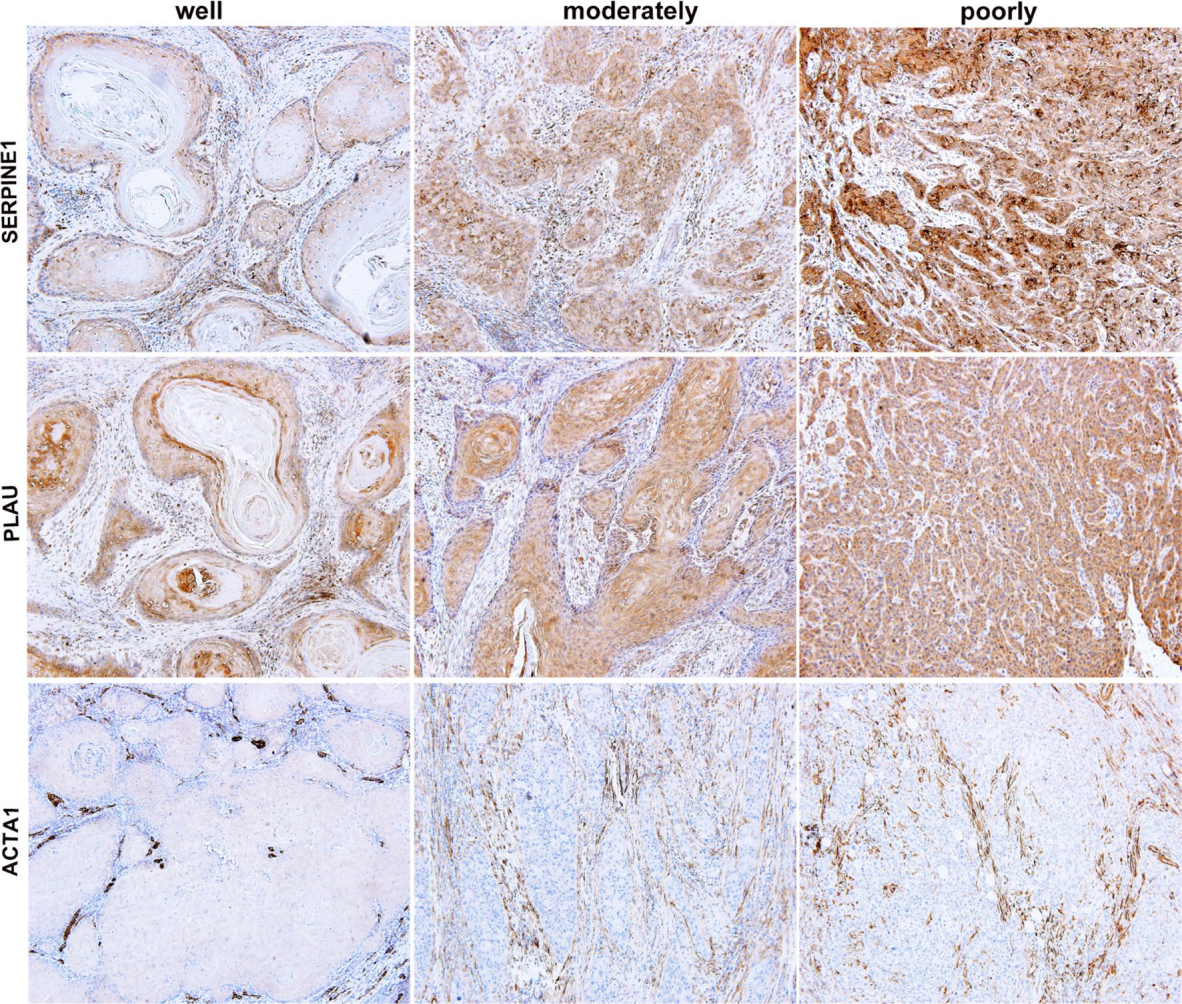
Immunohistochemical staining of SERPINE1, PLAU and ACTA1 in tumor nests of HNSCC tissues, and the expression levels of three genes were associated with tumor differentiation (× 100)

As the expression levels of SERPINE1, PLAU and ACTA1 were validated as independently predicted factors for OS of HNSCC patients, we continued to define the association of these genes and clinical histology classification in HNSCC samples. As shown in Fig. 6, there was a significant difference in these three hub genes expression between well and moderately or poorly differentiated HNSCC. The results showed that the LS of SERPINE1 increased significantly from well (166.78 ± 13.426%) through moderately (234.60 ± 36.439%) to poorly differentiated HNSCC samples (282.00 ± 7.589%) (Table 5). A significant difference in the LS of PLAU was also found between poorly and well (*p* = 0.000) or moderately differentiated HNSCC tissues (*p* = 0.005). In contrast, the expression of ACTA1 showed an obviously downward trend between well and moderately or poorly HNSCC samples; the mean LS of ACTA1 in well-differentiated HNSCC was 64.78 ± 9.400%; however, the results of LS between moderately and poorly differentiated HNSCC were all zero. Thus, our findings indicated that SERPINE1, PLAU and ACTA1 were correlated with clinical malignancy of HNSCC patients.

**Table 5:** The mean LS of SERPINE1, PLAU and ACTA1 in well, moderately and poorly differentiation of HNSCC tissues

Histology	Mean LS ± SD (100%)		
	SERPINE1	PLAU	ACTA1
Well	166.78 ± 13.426	106.78 ± 37.323	64.78 ± 9.400
Moderately	234.60 ± 36.439*	182.40 ± 6.450*	0.000*
Poorly	282.00 ± 7.589*^,^^#^	272.50 ± 36.812*^,#^	0.000*

*LS* labeling scores

*p < 0.05, compared to well-differentiated group; ^#^*p *< 0.05, compared to moderately differentiated group

## Discussion

Identifying oncogenic biomarkers and elucidating the underlying mechanism of the initiation and development of HNSCC would greatly benefit the early diagnosis and effective treatment for patients with high malignancy [17]. Emergency bioinformatics analysis has provided a powerful tool for the identification of biomarkers and therapeutic targets relevant to tumor progression and treatment response [18]. In the present study, we identified 52 up-regulated and 80 down-regulated DEGs through analyzing available data of gene expression profile datasets (GSE6631, GSE58911 and TCGA) in HNSCC by multiple bioinformatics tools. Functional analysis demonstrated that these DEGs are mainly associated with activation of ECM–receptor interaction and focal adhesion and suppression of drug metabolism–cytochrome P450 pathways. More importantly, based on TCGA dataset, our clinical experiments proved that a set of prognostic signatures including SERPINE1, PLAU and ACTA1 were identified as biomarkers for diagnosis and prognosis of HNSCC, which may provide novel insights for unraveling pathogenesis of HNSCC.

Recently, some basic studies have been conducted to identify the DEGs in HNSCC [19, 20]. For example, Yang et al. analyzed the gene expression profile of GSE6791 and identified 550 up-regulated and 261 down-regulated genes [21]. Similarly, Zhao found that PLAU, CLDN8 and CDKN2A could predict OS using gene expression profiles of GSE13601, GSE30784, GSE37991 and TCGA in oral squamous cell carcinoma [22]. Our integrated bioinformatics analysis indicated that 132 genes were consistently and significantly deregulated in GSE6631, GSE58911 and TCGA. Interestingly, our results revealed that there were also examples of genes that did not overlap compared with these reports; the main reason of this discrepancy may be because we used 3 different multiple profiles, which could greatly minimize the intra-tumoral heterogeneity and diversity of anatomical sites of HNSCC.

As was suggested by DAVID analysis, the up-regulated DEGs were mainly involved in extracellular matrix organization, collagen catabolic process, extracellular matrix disassembly, cell adhesion and collagen fibril organization at the level of BP. Extracellular matrix (ECM), as a crucial component of the cancer cell niche, provides the mechanical support for the tissue and mediates the cell–microenvironment interactions [23]. Significantly, collagens are one of the major proteins found within the ECM, and have themselves been implicated in many aspects of neoplastic transformation. Therefore, it is consistent with the findings that active functions of these cellular processes through ECM were the main cause for tumor development, progression and metastasis [24], whereas the down-regulated DEGs in HNSCC were mainly enriched in actin-mediated cell contraction and filament sliding, which were associated with decreased muscle function-mediated cytoskeleton remodeling in cancer development and progression [25]. Furthermore, the enriched KEGG pathway of up-regulated DEGs mainly induced ECM–receptor interaction, focal adhesion and PI3K–Akt signaling pathway. Significantly, 12 overlapping genes, including ITGA6, SPP1 and FN1, were identified to functionally involve in interactions between ECM and cells by activating these three signaling pathways, which lead to a direct or indirect control of cellular activities such as cell migration, differentiation and proliferation [26–28]. As a contrast, down-regulated DEGs were related to drug metabolism–cytochrome P450. The recent study has reported that the cytochrome P450 slowed metabolizers CYP2C9*2 and CYP2C9*3, which could directly regulate tumorigenesis via reduced epoxyeicosatrienoic acid production [29]. Together, these data suggested that deregulated pathways may be a major factor of HNSCC tumorigenesis, detecting these aberrant signaling pathways could precisely predict tumor progression [30].

After constructing PPI network with DEGs and listing the top degree of hub genes, the most significant two modules were filtered from the PPI network complex; consequent functional analysis showed that most of corresponding genes were associated with ECM–receptor interaction and focal adhesion. Furthermore, survival analysis of hub genes in these two modules revealed that SERPINE1, PLAU, ACTA1, MYL1, MYH2 and MYLPF were identified as prognostic markers for clinical outcome in the TCGA cohort. Among the up-regulated hub genes, PLAU, one of the major proteolytic enzymes involved in degradation of extracellular matrix, has been demonstrated to play critical roles in tissue remodeling and migration in the developmental as well as tumorigenesis process, whereas SERPINE1, as the most important physiological inhibitor of the PLAU, could in turn reverse this process and regulate the adhesion/ deadhesion balance of cells to the ECM [31]. However, it has been reported that SERPINE1 could induce the EMT process and promote tumor cell survival in breast and ovarian cancers [32, 33]. In our study, the bioinformatics analysis revealed significantly increased expressions of PLAU and SERPINE1 in HNSCC tissues, which were associated with poor clinical outcome. In contrast, for the down-regulated actin-family genes, ACTA1 gene encodes a protein exerting functions in cell motility, structure and integrity. Consistent with our observation, ACTA1 is also down-regulated in colorectal cancer [34]. In addition, our results showed that the other three specific down-regulation genes (MYL1, MYH2 and MYLPF) were involved in muscle contraction process, which might play a regulatory role in remodeling of muscle function in HNSCC tissues; however, the specific roles of these genes in cancers still need to be elucidated.

Of note, in view of the prognostic potency of these hub genes for HNSCC in TCGA database, by the validation of their top degree of genes and change levels of mRNA in microarrays, we selected SERPINE1, PLAU and ACTA1 to further detect their protein level by immunostaining. Our clinical analysis showed that SERPINE1, PLAU and ACTA1 were significantly changed in the progression of HNSCC. They were aberrantly expressed in the epithelium of OED and HNSCC and correlated with aggressiveness of HNSCC patients, which implied that these signature genes are possibly not only involved in the initiation of tumorigenesis but also late stages of cancer. Therefore, SERPINE1, PLAU and ACTA1 could be potentially utilized as diagnostic and prognostic biomarkers for HNSCC. More importantly, by comparing the extent of protein changes, the overexpressed SERPINE1 and PLAU are the most promising markers, and its detection could help to identify tumor cells in tissues.

In conclusion, the current study was intended to identify DEGs with comprehensive bioinformatics analysis to find the potential biomarkers and predict progression of HNSCC. We found that SERPINE1, PLAU and ACTA1 might be exploited as diagnostic and prognostic indicators for HNSCC. Finally, the results also suggested that the function of ECM–receptor interaction and focal adhesion may be essential signaling pathways in the development of HNSCC. Hence, our findings could significantly improve our understanding of the cause and underling molecular events of HNSCC, and provide potential targets for anticancer therapies.

## Electronic supplementary material

Below is the link to the electronic supplementary material.

Supplementary file1 (DOCX 34 kb)

Supplementary Figure 1. The expression changes of OS-associated genes (SERPINE1, PLAU, ACTA1, MYL1, MYH2 and MYLPF) in GSE6631, GSE58911 and TCGA datasets. FC, Fold change. (DOCX 8814 kb)

## References

[1] Torre LA, Bray F, Siegel RL (2015). Global cancer statistics, 2012. CA Cancer J Clin.

[2] Bray F, Ferlay J, Soerjomataram I et al (2018) Global cancer statistics 2018: GLOBOCAN estimates of incidence and mortality worldwide for 36 cancers in 185 countries. CA Cancer J Clin. https://orcid.org/10.3322/caac.21492 10.3322/caac.21492 30207593

[3] Miller KD, Siegel RL, Lin CC (2016). Cancer treatment and survivorship statistics, 2016. CA Cancer J Clin.

[4] Puram SV, Tirosh I, Parikh AS et al (2017) Single-cell transcriptomic analysis of primary and metastatic tumor ecosystems in head and neck cancer. Cell 171(7):1611 e162–1624 e162. 10.1016/j.cell.2017.10.044 PMC5878932 29198524

[5] Vervoort Y, Linares AG, Roncoroni M (2017). High-throughput system-wide engineering and screening for microbial biotechnology. Curr Opin Biotechnol.

[6] Kuang J, Zhao M, Li H (2016). Identification of potential therapeutic target genes and mechanisms in head and neck squamous cell carcinoma by bioinformatics analysis. Oncol Lett.

[7] Kuriakose MA, Chen WT, He ZM (2004). Selection and validation of differentially expressed genes in head and neck cancer. Cell Mol Life Sci CMLS.

[8] Lobert S, Graichen ME, Hamilton RD (2014). Prognostic biomarkers for HNSCC using quantitative real-time PCR and microarray analysis: beta-tubulin isotypes and the p53 interactome. Cytoskeleton.

[9] Deng M, Bragelmann J, Schultze JL (2016). Web-TCGA: an online platform for integrated analysis of molecular cancer data sets. BMC Bioinform.

[10] Gene Ontology C (2015). Gene ontology consortium: going forward. Nucleic Acids Res.

[11] Kanehisa M, Furumichi M, Tanabe M (2017). KEGG: new perspectives on genomes, pathways, diseases and drugs. Nucleic Acids Res.

[12] da Huang W, Sherman BT, Lempicki RA (2009). Systematic and integrative analysis of large gene lists using DAVID bioinformatics resources. Nat Protoc.

[14] Praneenararat T, Takagi T, Iwasaki W (2012). Integration of interactive, multi-scale network navigation approach with Cytoscape for functional genomics in the big data era. BMC Genom.

[15] Zhao T, Hu F, Qiao B (2015). Telomerase reverse transcriptase potentially promotes the progression of oral squamous cell carcinoma through induction of epithelial–mesenchymal transition. Int J Oncol.

[16] Zhao T, Hu F, Liu X (2015). Blockade of telomerase reverse transcriptase enhances chemosensitivity in head and neck cancers through inhibition of AKT/ERK signaling pathways. Oncotarget.

[17] Kang H, Kiess A, Chung CH (2015). Emerging biomarkers in head and neck cancer in the era of genomics. Nat Rev Clin Oncol.

[18] Hollstein M, Alexandrov LB, Wild CP (2017). Base changes in tumour DNA have the power to reveal the causes and evolution of cancer. Oncogene.

[19] Yan L, Zhan C, Wu J (2016). Expression profile analysis of head and neck squamous cell carcinomas using data from The Cancer Genome Atlas. Mol Med Rep.

[20] Li X, Sun R, Geng X (2017). A comprehensive analysis of candidate gene signatures in oral squamous cell carcinoma. Neoplasma.

[21] Yang B, Chen Z, Huang Y (2017). Identification of potential biomarkers and analysis of prognostic values in head and neck squamous cell carcinoma by bioinformatics analysis. OncoTargets Ther.

[22] Zhao X, Sun S, Zeng X (2018). Expression profiles analysis identifies a novel three-mRNA signature to predict overall survival in oral squamous cell carcinoma. Am J Cancer Res.

[23] He X, Lee B, Jiang Y (2016). Cell–ECM interactions in tumor invasion. Adv Exp Med Biol.

[25] Rubinstein B, Pinto IM (2015). Epithelia migration: a spatiotemporal interplay between contraction and adhesion. Cell Adhes Migr.

[26] Brooks DL, Schwab LP, Krutilina R (2016). ITGA6 is directly regulated by hypoxia-inducible factors and enriches for cancer stem cell activity and invasion in metastatic breast cancer models. Mol Cancer.

[27] Wu J, Wang Y, Xu X (2016). Transcriptional activation of FN1 and IL11 by HMGA2 promotes the malignant behavior of colorectal cancer. Carcinogenesis.

[28] Xu C, Sun L, Jiang C (2017). SPP1, analyzed by bioinformatics methods, promotes the metastasis in colorectal cancer by activating EMT pathway. Biomed Pharmacother Biomed Pharmacother.

[29] Sausville LN, Gangadhariah MH, Chiusa M (2018). The cytochrome P450 slow metabolizers CYP2C9*2 and CYP2C9*3 directly regulate tumorigenesis via reduced epoxyeicosatrienoic acid production. Cancer Res.

[30] Isaacsson Velho PH, Castro G Jr, Chung CH (2015) Targeting the PI3K pathway in head and neck squamous cell carcinoma. Am Soc Clin Oncol Educ Book ASCO Am Soc Clin Oncol Meet. 10.14694/EdBook_AM.2015.35.123 25993150

[31] Pavon MA, Arroyo-Solera I, Cespedes MV (2016). uPA/uPAR and SERPINE1 in head and neck cancer: role in tumor resistance, metastasis, prognosis and therapy. Oncotarget.

[32] Azimi I, Petersen RM, Thompson EW (2017). Hypoxia-induced reactive oxygen species mediate *N*-cadherin and SERPINE1 expression, EGFR signalling and motility in MDA-MB-468 breast cancer cells. Sci Rep.

[33] Pan JX, Qu F, Wang FF (2017). Aberrant SERPINE1 DNA methylation is involved in carboplatin induced epithelial–mesenchymal transition in epithelial ovarian cancer. Arch Gynecol Obstet.

[34] Liu J, Li H, Sun L (2017). Aberrantly methylated-differentially expressed genes and pathways in colorectal cancer. Cancer Cell Int.

[13] Szklarczyk D, Franceschini A, Wyder S (2015). STRING v10: protein–protein interaction networks, integrated over the tree of life. Nucleic Acids Res.

[24] Multhaupt HA, Leitinger B, Gullberg D (2016). Extracellular matrix component signaling in cancer. Adv Drug Deliv Rev.

