# Supplementary material for: Identification of SERPINE1, PLAU and ACTA1 as biomarkers of head and neck squamous cell carcinoma based on integrated bioinformatics analysis
Source: Int J Clin Oncol. 2019 Apr 1;24(9):1030–41. doi: 10.1007/s10147-019-01435-9 (PMC6687676; doi:10.1007/s10147-019-01435-9)
Supplement: Supplementary file 1 — Supplementary file1 (DOCX 34 kb) [file 10147_2019_1435_MOESM1_ESM.docx]

Supplementary Table 1. Human HNSCC sample information.

| Sample | Gender | Age | Site | TNM | Differentiation |
| --- | --- | --- | --- | --- | --- |
| 1 | F | 53 | Tongue | T2N2bM0 | moderately |
| 2 | M | 57 | Tongue | T2N2bM0 | well |
| 3 | F | 78 | Gingiva | T2N0M0 | well |
| 4 | M | 74 | Oropharynx | T2N0M0 | moderately |
| 5 | F | 52 | Gingiva | T1N0M0 | well |
| 6 | F | 57 | Bucca | T1N0M0 | moderately |
| 7 | M | 52 | Oropharynx | T2N0M0 | moderately |
| 8 | F | 51 | Gingiva | T1N0M0 | well |
| 9 | F | 59 | Tongue | T2N2bM0 | well |
| 10 | F | 81 | Palate | T2N0M0 | well |
| 11 | M | 58 | Bucca | T1N0M0 | moderately |
| 12 | F | 63 | Oropharynx | T3N0M0 | moderately |
| 13 | F | 54 | Gingiva | T3N0M0 | well |
| 14 | F | 43 | Tongue | T1N2aM0 | poorly |
| 15 | F | 66 | Gingiva | T2N1M0 | well |
| 16 | F | 50 | Tongue | T1N0M0 | poorly |
| 17 | F | 65 | Tongue | T2N1M0 | well |
| 18 | M | 76 | Gingiva | T2N0M0 | moderately |
| 19 | M | 62 | Bucca | T2N2aM0 | moderately |
| 20 | M | 36 | Tongue | T1N1M0 | well |
| 21 | F | 72 | Tongue | T2N2aM0 | moderately |
| 22 | M | 60 | Tongue | T2N0M0 | well |
| 23 | M | 61 | Oropharynx | T1N1M0 | well |
| 24 | M | 34 | Tongue | T1N0M0 | well |
| 25 | M | 66 | Bucca | T1N0M0 | well |
| 26 | M | 51 | Tongue | T1N0M0 | well |
| 27 | F | 67 | Tongue | T1N0M0 | poorly |
| 28 | M | 70 | Bucca | T2N1M0 | poorly |
| 29 | M | 56 | Palate | T1NOM0 | well |
| 30 | M | 72 | Gingiva | T2N0M0 | well |
| 31 | F | 38 | Tongue | T1N2aM0 | well |
| 32 | M | 54 | Oropharynx | T2N2cM0 | poorly |
| 33 | M | 47 | Bucca | T2N2cM0 | poorly |
| 34 | F | 52 | Gingiva | T2N0M0 | well |
| 35 | M | 72 | Gingiva | T2N0M0 | moderately |
| 36 | M | 72 | Oropharynx | T1N0M0 | well |
| 37 | F | 64 | Gingiva | T2N0M0 | well |
| 38 | F | 47 | Tongue | T2N2aM0 | well |
| 39 | F | 42 | Tongue | T2N0M0 | well |

All head and neck tumor samples examined in the study were clinically diagnosed and confirmed as squamous cell carcinoma.
