# Supplementary figures and images for: Identification of SERPINE1, PLAU and ACTA1 as biomarkers of head and neck squamous cell carcinoma based on integrated bioinformatics analysis
Source: Int J Clin Oncol. 2019 Apr 1;24(9):1030–41. doi: 10.1007/s10147-019-01435-9 (PMC6687676; doi:10.1007/s10147-019-01435-9)

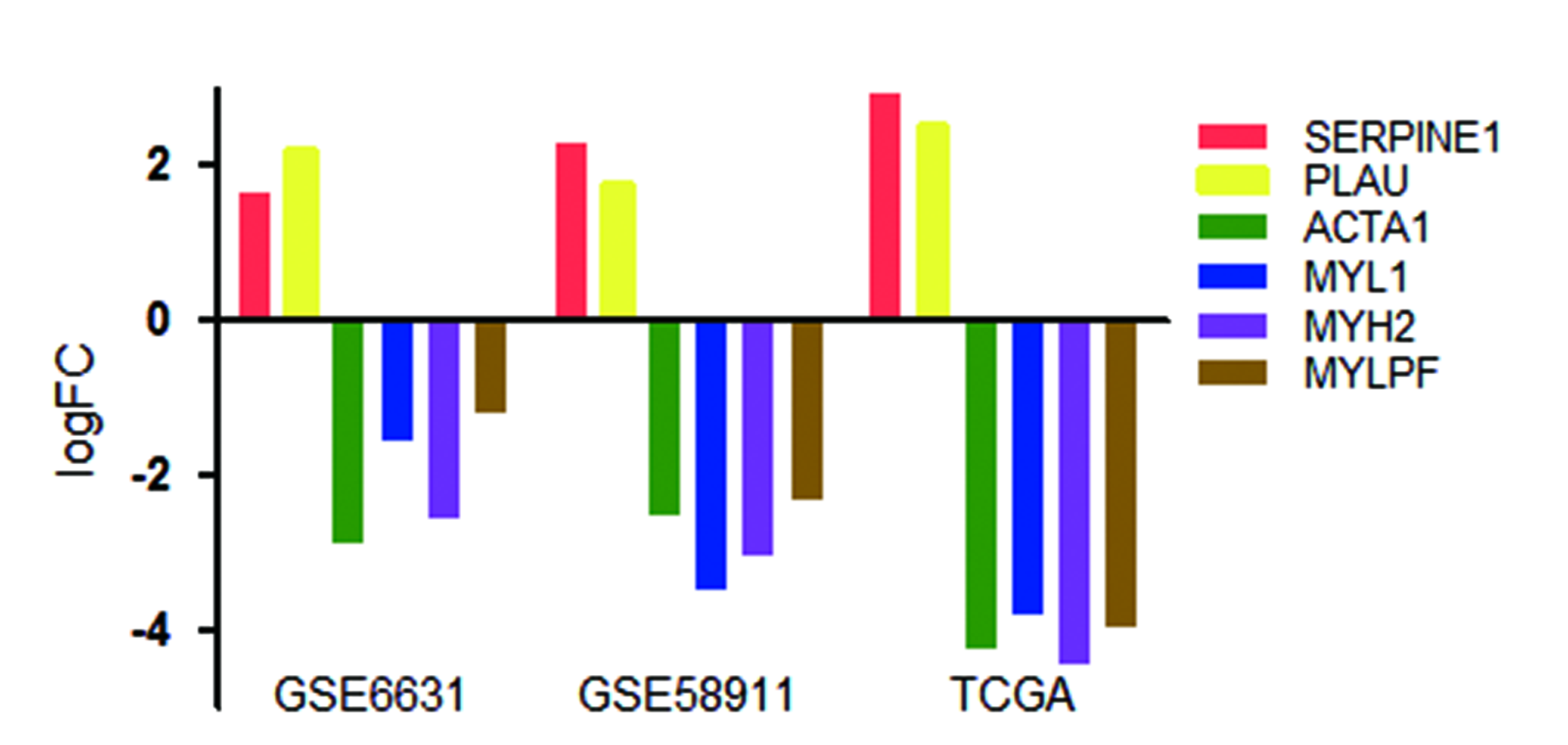

Supplement: Supplementary file 2 — Supplementary Figure 1. The expression changes of OS-associated genes (SERPINE1, PLAU, ACTA1, MYL1, MYH2 and MYLPF) in GSE6631, GSE58911 and TCGA datasets. FC, Fold change. (DOCX 8814 kb) [file 10147_2019_1435_MOESM2_ESM.docx]
